# Depression as a cardiovascular disorder: central-autonomic network, brain-heart axis, and vagal perspectives of low mood

**DOI:** 10.3389/fnetp.2023.1125495

**Published:** 2023-05-16

**Authors:** Gaetano Valenza

**Affiliations:** NeuroCardiovascular Intelligence Lab, Bioengineering and Robotics Research Center E Piaggio & Department of Information Engineering, University of Pisa, Pisa, Italy

**Keywords:** heart rate variabiity (HRV), central autonomic network (CAN), depression, vagal activity, sympathetic activity, brain-heart interplay, brain-heart axis

## Abstract

If depressive symptoms are not caused by the physiological effects of a substance or other medical or neurological conditions, they are generally classified as mental disorders that target the central nervous system. However, recent evidence suggests that peripheral neural dynamics on cardiovascular control play a causal role in regulating and processing emotions. In this perspective, we explore the dynamics of the Central-Autonomic Network (CAN) and related brain-heart interplay (BHI), highlighting their psychophysiological correlates and clinical symptoms of depression. Thus, we suggest that depression may arise from dysregulated cardiac vagal and sympathovagal dynamics that lead to CAN and BHI dysfunctions. Therefore, treatments for depression should target the nervous system as a whole, with particular emphasis on regulating vagal and BHI dynamics.

## 1 Introduction

The World Health Organization (WHO) reports that “*depression is a leading cause of disability worldwide*” and the condition affects at least 322 million people. Even more surprising is that 10 million people with depression have thought about suicide and 3 million have actual suicide plans. Those who actually seek help are diagnosed through the administration of questionnaires and subjective interviews. To date no specific physiological or biochemical markers are considered in current clinical practice, and no physiological measurement objectively distinguishes among the different subtypes of depression. Economically-speaking, the cost of lost productivity due to depression in the EU has been estimated at over €70 billion per year (World Health Organization Regional Office for Europe, 2018; Jaffe et al., 2019; Johnston et al., 2019; Greenberg et al., 2021).

According to the Diagnostic and Statistical Manual of Mental Disorders Fifth Edition (DSM-5 American Psychiatric Association, DSM-5 Task Force, 2013), if depressive symptoms are not attributable to the physiological effects of a substance or other medical or neurological conditions, the depression and related subtypes are to be considered *mental* disorders, i.e., disorders somehow targeting the central nervous system (CNS) exclusively.

But why do we assume that these changes result from intrinsic functioning of the brain only? Why is it that more than 60% of patients with major depression may show no response to first-line antidepressant treatment mainly targeting brain neurotransmitters (Johnston et al., 2019)? Why do recent meta-analyses show inconsistency between studies that only focus on brain dynamics and depression (e.g., Müller et al., 2017)? These questions suggest that previous literature on depression has mainly focused on brain dynamics, while giving inadequate attention to body dynamics.

The brain and heart have long been studied and treated individually at the cortical/subcortical or neuro-peripheral level, including cardiovascular, blood pressure and respiration dynamics (as part of non-invasive monitoring) using specific techniques focused on specific system dynamics. However, the physiological system is complex. In other words, because of the numerous interactions of many subcomponents, the system as a whole exhibits characteristics that the individual components cannot act on. The CNS and autonomic nervous system (ANS) control all body organs simultaneously through anatomical and biochemical/functional connections that have a lasting impact on health and especially disease.

In piecing together segregated parts of research and in following the development of a comprehensive vision on whole-nervous system mental health and related science, here we pose the following fundamental hypotheses:

- In the absence of medical or neurological illnesses, brain changes in depression are not only the cause but also the consequence of a mood disorder
- The nervous system as a whole mediates emotional processing and regulation
- Abnormal vagal activity propels emotion dysregulation in depression

Scientific evidence supporting these hypotheses leverage upon the definition of the Central-Autonomic Network (CAN), which comprises brain regions involved in autonomic control (Benarroch, 1993; Beissner et al., 2013; Valenza et al., 2019; Valenza et al., 2020), as well as Brain-Heart Interplay (BHI), which comprises the functional links between CNS and ANS through electrical, biochemical, and physical communications. In this perspective, depression is envisioned as a manifestation of a dysregulation of cardiac vagal and sympathovagal dynamics sustaining CAN dysfunctions, which in turn reflects on functional BHI. Consequently, a “functional-vagal theory” of depression is stated, suggesting that depression treatment should also target brain-heart dynamics and, especially, act at a nervous-system-wise level.

### 1.1 Central and autonomic correlates of emotion regulation and emotional processing at a glance

Emotion regulation refers to the process by which individuals modify, maintain, or control their emotions, including their experience, expression, and physiological responses (Gross, 2015). It involves a range of strategies, such as cognitive reappraisal, expressive suppression, and attentional deployment, that are used to regulate emotional experiences and their impact on behavior and cognition. Emotion regulation can be adaptive, promoting psychological wellbeing and social functioning, or maladaptive, contributing to psychopathology and interpersonal difficulties. On the other hand, emotional processing refers to the cognitive and affective operations involved in the appraisal, interpretation, and response to emotional stimuli (Hamann and Canli, 2004). It includes perceptual, attentional, and memory processes, as well as higher-order cognitive processes, such as cognitive reappraisal and problem-solving. Emotional processing can be influenced by individual differences in emotion regulation and emotion dysregulation, as well as by cultural and social factors.

The vagus nerve, a complex network of nerve fibers that originates in the brainstem and extends throughout the body, is known to be involved in a wide range of physiological processes, including emotion regulation, processing, and communication (Porges, 2007; Rottenberg, 2007). Different vagal control systems seem to be phylogenetically ordered and behaviorally linked to social communication, mobilization and immobilization (Porges, 2007). Activation of the ANS during encoding or retrieval of emotional information may modulate the neural processes mediating mood congruent memory (Critchley et al., 2003; 2005). Neural control of the heartbeat as measured through Heart Rate Variability (HRV) is involved in processing of emotional information (for example, Valenza and Scilingo, 2014; Damasio et al., 2000; Panksepe, 2000; Lewis et al., 2010), and regulation of brain activity dynamics (Craig, 2003; Lane et al., 2009; Thayer et al., 2012; Beissner et al., 2013).

At a nervous-system-wise level, the brain and the ANS concurrently regulate all peripheral systems and modulate emotional regulation and processing, as well as mood states (Craig, 2003; Lane et al., 2009; Lane and Wager, 2009; Thayer et al., 2012; Beissner et al., 2013; Candia-Rivera et al., 2022). Functionally, emotions have been linked to the brain’s predictions about the state of the body. The predictions are constantly compared to ascending bodily or interoceptive signals, transmitted, for example, by the vagus nerve (Candia-Rivera et al., 2022; Hsueh et al., 2023). If the resulting prediction error, that is, the difference between the predicted and the actual state, is too big, subjective (e.g., affective) consequences ensue in order to re-balance disturbances of homeostasis, for example, by adaptive behaviour (Seth, 2013). Such interoceptive predictions, which have also been related to depression (Barrett and Simmons, 2015), are reflected in the interaction between the CNS and the ANS (Candia-Rivera et al., 2022; Hsueh et al., 2023).

### 1.2 Depression, subtypes, and somatic symptoms

Depression is a common disorder characterized by persistent feelings of sadness, loss of interest or pleasure in activities, and other symptoms that impair daily functioning (e.g., feelings of guilt or low self-worth, disturbed sleep or appetite, feelings of tiredness, poor concentration). Depression is associated with a range of cognitive, affective, and behavioral deficits, including difficulties in emotion regulation and emotion processing. Individuals with depression often experience emotion dysregulation, characterized by heightened negative affect, rumination, and reduced positive affect. Indeed, emotion dysregulation, refers to difficulties in regulating emotions effectively. Emotion dysregulation can manifest as intense, unstable, or inappropriate emotional responses, or as difficulties in modifying emotional responses to fit the situation (Abravanel and Sinha, 2015).

In 2008, WHO globally ranked major depression as the third cause of burden of disease and projected that the disease will rank first by 2030 (Malhi and Mann, 2018), unmasking social and economic costs of depressed state (Olesen et al., 2012). Moreover, despite the progress in both pharmacological and psychological therapies, clinicians involved in the management of depression are often faced with treatment resistance, highlighting the necessity to develop alternative therapeutic options. Recent research also suggests that depression symptoms cycle with daily rhythms and hormonal changes (Mendoza, 2019), but there is currently no technology to measure objective biomarkers in daily life. Current diagnosis relies on “structured” interviews based on a patient’s subjective description of symptoms and subsequent interpretation of these by a physician. Diagnosis of major depression results from a positive response to 5 out of 9 listed symptoms, many of which are total opposites of each other (DSM-5). Having a wide variation of symptoms and behaviour, severity, onset and course, depression is a very *heterogeneous* disorder (Lux and Kendler, 2010; Goldberg, 2011; Lieblich et al., 2015; Drysdale et al., 2017; Feczko et al., 2019). While behavioural correlates of depression are reported in DSM-5 2013, clinically-reliable physiological and biochemical markers for an objective diagnosis are unknown despite encouraging research findings (Rottenberg, 2007; Corrigan et al., 2010; Gaebler et al., 2013; Valenza and Scilingo, 2014; Garcia et al., 2016; Drysdale et al., 2017; Gentili et al., 2017; Brown et al., 2018; Caldwell and Steffen, 2018; Hartmann et al., 2019; Catrambone et al., 2021).

Although several studies have described different subtypes of depression (van Loo et al., 2012; Kessing and Bukh, 2017; Rantala et al., 2018; Beijers et al., 2019), the etiology and etiological factors involved have rarely been studied (Kessing and Bukh, 2017). Clinically, subtypes of depression are important for predicting prognosis and treatment outcome and can be identified by polarity, symptoms, onset (due to a particular event, season or age), recurrence and severity (Thase, 2013). In terms of bipolarity, a distinction is made between unipolar depression and bipolar depression, where bipolar depression is characterized by mood changes, including (hypo) mania and/or mixed episodes. The DSM-5 American Psychiatric Association, DSM-5 Task Force, 2013 distinguishes four symptom profiles: depression, atypical, anxiety, and psychotic depression. Major depression is characterized by three or more symptoms, including anhedonia or lack of a mood response to positive events, psychomotor inhibition or agitation, weight loss, excessive guilt, and trouble sleeping early in the morning. Major depression affects about 25%–30% of people with depression. People with depression usually do not respond to placebo treatment and may not benefit from psychotherapy and social interventions. Atypical depression features symptoms such as overeating, weight gain, and excessive sleeping, as well as sensitivity to phobias, anxiety, chronic pain, and rejection. Atypical patients generally have a higher proportion of younger women and higher rates of suicide attempts. Anxious depression is associated with abnormal anxiety, fear of tension or loss of control, while psychotic depression presents with delusions or hallucinations with a high relapse rate and frequent hospitalizations in 15%–20% of patients. In terms of onset, early-onset depression (ages 18–30) is more likely to present with personality disorders and neuroticism than late-onset depression (ages 31–70). DSM-5 differentiates mild, moderate, and severe depression based on severity, which predicts long-term risk of relapse and suicide and guides treatment options to some extent (e.g., mild depression cannot be treated with antidepressants). There is no chronic evolution of depressive episodes). Major depression involves an inadequate response to multiple treatments, is complicated by psychotic symptoms, and/or is associated with severe psychotic comorbidities or psychosocial factors (Clarkin et al., 2019). It affects approximately 30% of patients with major depression and there is no consensus on the biological basis (Rush et al., 2006; Fekadu et al., 2009; Trevino et al., 2014). Based on the latent class analysis model, three subtypes of treatment-resistant depression were identified. Major depression (frequency: 66%), moderate depression with anxiety (9%) and mild depression with anxiety/somatization (25%) (Liao et al., 2019).

According to National Institutes of Health and Caregiving guidelines, treatment of depression should include psychological education, low- or high-intensity psychosocial interventions, electroconvulsive therapy, and antidepressant treatment for severe depression. Although first-line pharmacological treatment for major depression usually consists of a selective serotonin reuptake inhibitor or a serotonin-norepinephrine reuptake inhibitor as monotherapy, up to 50%–60% of patients with major depression may not respond to the initial treatment and may require alternative therapies or combination treatments (Johnston et al., 2019). Furthermore, there is no evidence that serotonin transporter genotype alone or its interaction with stressful life events is associated with an increased risk of depression (Risch et al., 2009). Lifestyle habits, including diet, exercise and sleep, have been shown to play an important role (Lopresti et al., 2013; Kaseva et al., 2016). Although mainly targeting the brain, clinical procedures in the diagnosis and treatment of depression are already rooted in somatic interventions. The classification of depression subtypes is based on corporeal changes, such as weight loss/gain, and types of movements (retardation/agitation). Specific antidepressant therapies are also chosen based on their effects on *somatic* symptoms including constipation, nausea, and fatigue, e.g., a patient with nausea will be treated with mirtazapine rather than another serotoninergic drug (NICE National Institute for Health and Care Excellence, 2018). In the treatment of depressive symptoms, the so-called “implicit memory,” present in terms of somatic and affective states without awareness of connections with past experiences, is fundamental for trauma-focus psychotherapeutic treatment (Hase et al., 2017).

### 1.3 Vagal activity correlates of depression and depressive symptoms

Dysfunctional cardiac vagal activity has been increasingly linked to depression, substantiating the notion that a compromised ANS dynamics can contribute to mood disorders. Reduced cardiac vagal control, as measured through HRV series (Rajendra Acharya et al., 2006) is associated with depressive symptoms (Rottenberg, 2007; Kemp et al., 2010; Paniccia et al., 2017; Brown et al., 2018; Caldwell and Steffen, 2018; Hartmann et al., 2019). Reduced vagal activity in major depression was not only associated with depression severity but also predicted the persistence of depressive symptoms (Rottenberg, 2007). In contrast, patients with depression may exhibit increased vagal activity compared to healthy controls when experiencing heightened emotional reactivity in response to emotionally arousing stimuli (Garcia et al., 2016); the observed inconsistency may be attributed to the significant variability in the psychopathology of depressive symptoms among individuals, as well as the influence of different medication types and dosages on vagal activity (Rottenberg, 2007).

Depression may also lead to somatic diseases such as stroke, diabetes, and obesity, which are related to dysfunctions in metabolism, immunity, inflammation, and autonomic regulation (Penninnx et al., 2013). Moreover, dysfunctional vagal activity in depression may often result in heart disease (e.g., Rovai et al., 2015). A meta-analysis showed that depression increased the risk of all strokes by 34%–63% (Dong et al., 2012) and increased the risk by 30%–90% of the population with coronary artery disease (CAD) (Nicholson et al., 2006). Furthermore, the presence of depressive symptoms predicts a worse prognosis for CHD (Van Melle et al., 2004). Conversely, patients with CHD are more likely to develop depressive symptoms or overall major depressive disorder (Konrad et al., 2016). Thus, targeting depressive symptoms not only improves mood but also has a positive effect on CVD outcome (e.g., Angermann et al., 2016).

In line with the aforementioned evidence, vagus nerve stimulation has recently been approved by the EU regulatory body for treatment-resistant depression, supported by promising evidence (e.g., Kumar et al., 2019). In fact, non-invasive stimulation of the vagus nerve improves emotional regulation, confirming that the vagus nerve is causally involved in emotional processing (Colzato et al., 2017; Candia-Rivera et al., 2022; Hsueh et al., 2023).

Evidence on dysfunctional CAN and related BHI dynamics has been reported for depression and emotion dysregulation (Taggart et al., 2011; Terhaar et al., 2012; Gaebler et al., 2013; Gaebler et al., 2016; Catrambone et al., 2019).

In summary, depression is linked to dysfunctional vagal activity, which could potentially contribute to the development of somatic diseases. On one hand, depression might be related to parasympathetic-dominant hypoarousal, leading to diminished emotional expression, sensations of emptiness, helplessness, and hopelessness, excessive drowsiness, cognitive impairments, and weakened defensive responses. On the other hand, depression may be connected to decreased vagal activity levels (Chambers and Allen, 2002), although the findings are not entirely consistent (Rottenberg, 2007).

## 2 A functional-vagal theory and central-autonomic network perspectives

The aforementioned literature proves that there is a close association between emotional regulation and processing, ANS and CNS control over the body’s internal state. Nonetheless, it seems that contemporary psychiatry and clinical psychology largely assume that the functional and dynamic interactions within the human nervous system during emotional processing, regulation, and dysregulation are primarily driven by brain-based processes rather than by bottom-up processes, such as depression and its various subtypes. To this extent, it is worth mentioning the scientific debate on the nature of emotions that has lasted over a century (James, 1880). Recent research shows that neural control over heartbeat dynamics creates and initiates emotional responses (Candia-Rivera et al., 2022; Hsueh et al., 2023). This scientific finding undermines the “classical” emotion theories that suggest emotions are solely functional states of the brain. Consequently, the current grand challenge is to demonstrate the significant causal involvement of dynamical vagal and sympathovagal activities in depression and its subtypes, which may be reflected in changes in the brain.

*So why are vagal activity levels lower when a patient has parasympathetic-dominant hypoarousal symptoms?*

**FIGURE 1:**
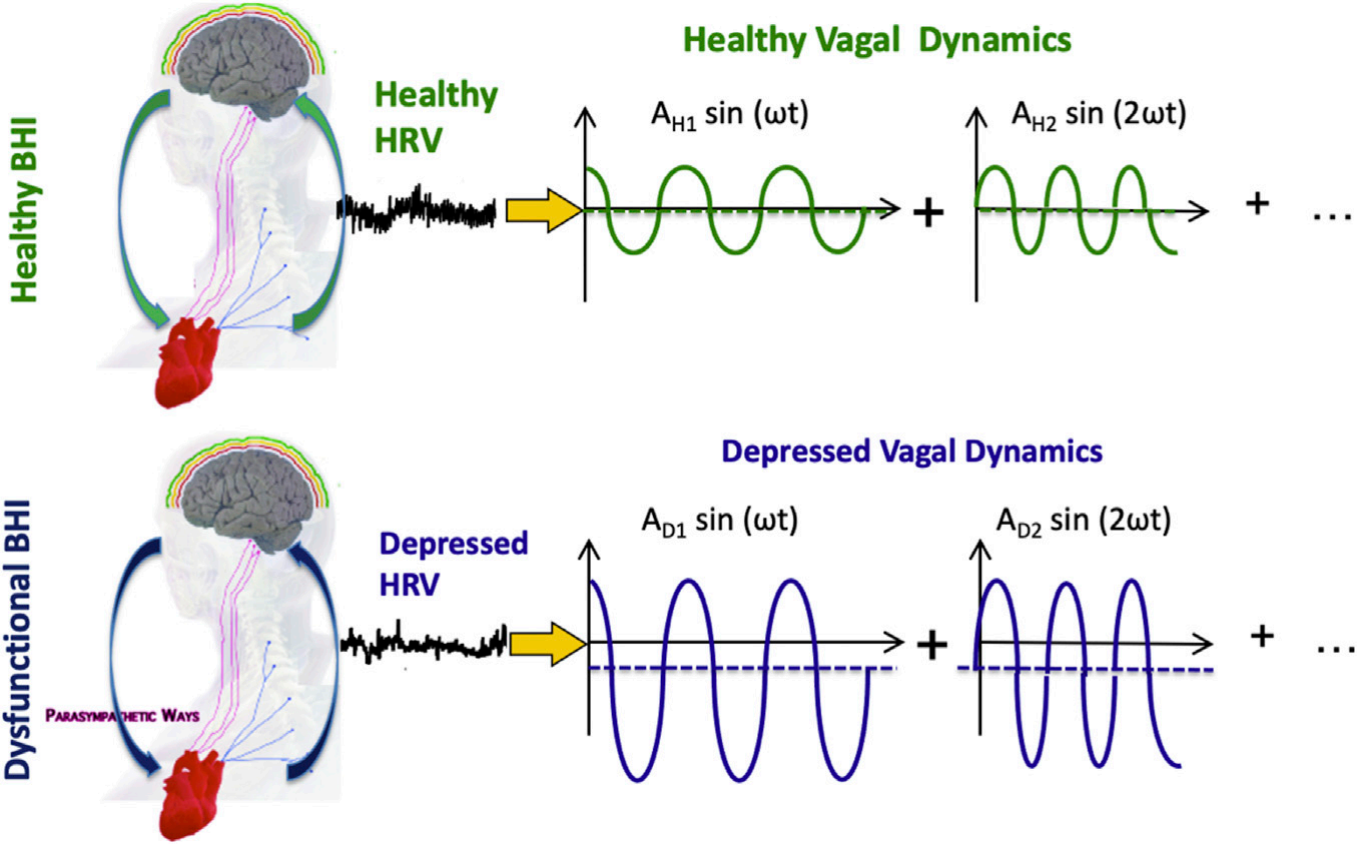
Functional BHI, HRV series, and vagal activity in exemplary healthy and depressed conditions. Vagal activity is illustrated in the Fourier domain, so each sinusoid represents a component of vagal oscillations. The mean of each sinusoid is linked to the so-called “vagal tone,” which may be reduced in depression. The amplitude of each sinusoid is thought to be linked to vagally-dominant hypoarousing symptoms in depression.

To explain the autonomic correlates observed in depression, it is here assumed that depression is a disease involving the parasympathetic nervous system, therefore involving dysregulation of the dynamical CAN through vagal control and sympathovagal interplay. Accordingly, let us assume that time-varying vagal activity results from the complex interaction between body- and brain-related dynamics, and pathological ANS dysfunctions are associated with significant CAN changes and emotional dysregulation. According to the *Fourier* theory, every time series can be represented as a linear combination (i.e., sums and differences) of sinusoidal functions that have specific amplitude and frequency. Figure 1.

Depression is often characterized by a similar or reduced vagal tone and increased emotional reactivity compared to healthy individuals, coupled with parasympathetic-dominant hypoarousal symptomatology. In any case, depression may be associated with pathological vagal dynamics. In order to transition from pathological vagal dynamics to healthy ones, the amplitude of the sinusoid, i.e., the emotional reactivity (Garcia et al., 2016), must be reduced, and the mean of the sinusoid, i.e., the vagal tone, must be increased. This approach is hereby referred to as the “*functional vagal theory of depression*” While this theory may be useful in understanding the underlying pathophysiology of depression and its relationship to neurocardiovascular dysfunctions, the non-specificity of brain and neurocardiovascular dynamics (Saul and Valenza, 2021), along with the nonlinear and complex nature of the cardiovascular system, makes its direct translational clinical application challenging.

Indeed, although numerous physiological conditions and pathologies are associated with HRV-derived and brain-signal-derived biomarkers that correlate with either the severity of the pathology or the physiological state, these correlations may not be particularly specific, especially when experimental conditions and methodological approaches vary. Consequently, it is possible that a healthy individual in a particular neuro-autonomic state may exhibit similar dynamics to a subject with pathology in a different psycho-physiological state. The nonlinearity of the cardiovascular system poses challenges since an increase in vagal activity could potentially result in an increase in heart rate, as the effect of vagal stimulation on heart rate strongly depends on the level of sympathetic stimulation occurring concurrently (Sunagawa et al., 1998). This phenomenon, known as accentuated antagonism, is partly attributed to the inhibitory effect of the vagally released ACh on the release of NorEpinephrine from nearby sympathetic nerve endings. Additionally, the complexity of the cardiovascular system, resulting from the many interactions of numerous sub-components, means that the system as a whole exhibits properties that the individual components acting alone cannot demonstrate. The system’s nonlinearity, coupled with the multiple feedback mechanisms of sympathetic and vagal activity on cardiovascular control, makes the system extremely sensitive to input, even infinitesimal changes (Sunagawa et al., 1998). Therefore, we do not explicitly endorse the use of the mean of the sinusoid, i.e., the so-called vagal tone, to detect depression. Rather, it is the author’s opinion that brain-heart related biomarkers are more promising in targeting specific oscillations and spatial information over the scalp or brain region associated with afferent or efferent peripheral activity (Pfurtscheller et al., 2017; Catrambone et al., 2021; Pfurtscheller et al., 2021; Pfurtscheller et al., 2022; Pfurtscheller et al., 2023).

Prospectively, the diagnosis and treatment of depression and its subtypes should move from a symptomatic- and brain-centred view exclusively to a functional and whole-body framework where vagally-mediated CAN dynamics play a crucial and causal role. In other words, modern medicine for this highly disabling mood disorder should reverse the current diagnostic and treatment framework for depression by moving from a symptomatic-only and brain-dominated view to a new functional-vagal and whole-body conception. To this extent, science needs to achieve the following goals:

*Gain Fundamental Knowledge on SympathoVagal activity & Functional Brain-Body interplay.* There is the need to develop effective biomarkers of *dynamical* sympathovagal activity and associated CAN/brain-heart interplay to characterize physiological sympathovagal activity and functional BHI in healthy conditions. These biomarkers may be defined from generic multivariate signal processing methods and subsequent feature extraction in the time, frequency, and/or nonlinear and complexity domains; *Characterise brain-heart-mediated emotional responses.* There is the need to characterise a dynamical emotional profile in the healthy through time-varying autonomic- and CAN-related metrics. Gender, age, and other socio-demographic factors should be considered because they are known to affect heartbeat dynamics and, therefore, CAN-related and BHI-related metrics; *Characterise brain-heart-mediated biomarkers specific of emotional dysregulation and depression.* An impaired dynamical emotional profile of depression should then be characterised throughout treatment by mapping and comparing associated autonomic and CAN and BHI patterns to a healthy profile. Experimental paradigms including standardised emotional elicitation as well as personalised recall scripts combined with non-invasive brain stimulation should be employed to stimulate the emergence of dysfunctional emotion regulation. The desired outcome may be achieved through identifying personalized autonomic and BHI-related features that are distinct to each individual and contribute to their depressive symptomatology. By utilizing these features, the pace and intensity of brain and vagal stimulations can be adjusted accordingly, enabling concurrent progress towards remission and recovery.

Taking a functional-vagal perspective and a broader view of the nervous system into account can help clinicians better understand the clinical symptoms of depression that drive current practice and allow for objective neurophysiological assessment. Instead of changing the current clinical paradigm, this approach can complement the existing diagnostic framework and provide new diagnostic criteria that take into account the essential role of aetiology and pathophysiology in diagnostic decision-making. By adopting quantitative assessments based on specific BHI-related disease biomarkers, modern psychiatry can align itself with other medical specialties, including cardiology and neurology. This approach will enable psychiatry to achieve a more comprehensive understanding of the biological underpinnings of mood disorders, bringing it closer to the diagnostic and treatment practices of these related fields. This holistic view may contribute to the update of the so-called Research Domain Criteria (Morris and Cuthbert, 2012), which proposes to study five psychopathological domains including negative and positive valence, cognitive systems, social processes, and arousal/regulatory systems to better understand mood disorders.

It is important to note that a comprehensive investigation of dysfunctional neural dynamics in depression should also consider sympathetic dynamics. In terms of symptomatology, sympathetically dominated hyperarousal is marked by emotional hyperactivity, reactivity, impulsivity, anxiety, and anger, accompanied by parasympathetic hyposensitivity. Evidence concerning the correlation between sympathetic activity and depression is relatively scarce compared to vagally-related measures, likely due to challenges in quantitatively assessing sympathetic activity (Valenza et al., 2018). Nevertheless, it is posited that dysfunctional sympathetic dynamics may contribute to the increased risk of cardiac complications observed in depression (Nicholson et al., 2006; Barton et al., 2007; Dong et al., 2012). Indeed, exposure to risk or trauma has been found to stimulate the ANS, resulting in sympathetic and/or parasympathetic hyperalertness, leading to a pathological mood state in which an individual is unable to experience positive emotional states (Corrigan et al., 2010). When uncontrolled ANS (i.e., sympathetic-dominant hyperarousal and/or parasympathetic-dominant hypoarousal) cannot control heightened emotional or depressive states, patients often report being unable to cope with emotional and physiological arousal (Ogden, 2006; Corrigan et al., 2010). The “window of tollerance” model of autonomic arousal states that there is a “window” of healthy autonomic tolerance when intense emotions and a state of calm or relaxation can be integrated and integrated throughout the body. The periaqueductal grey area, which is also part of the CAN, is thought to be involved in these mechanisms (Corrigan et al., 2010; Beissner et al., 2013). Identifying CNS-ANS or brain-heart markers of depression—ideally under naturalistic conditions—can help maintain or re-establish a healthy “Window of Tolerance,” for example, through personalised concurrent brain-body stimulation.

Considering the swiftly growing body of scientific evidence highlighting dysfunctional BHI dynamics in a range of pathological conditions (Taggart et al., 2011; Tahsili-Fahadan et al., 2017; Joyner, 2019; Riching et al., 2019; Riganello et al., 2019; Liu et al., 2020; Méloux et al., 2020; Costagliola et al., 2021; Nowroozpoor et al., 2021; Tumati et al., 2021; Vaccarino et al., 2021; Bekała et al., 2022; Grassi et al., 2022; Kumral et al., 2022; Lazaridi et al., 2022; Liu et al., 2022; Seligowski et al., 2022; Wang and Peng, 2022; Xue et al., 2022), it is possible that cardiologists will run EEG/psychometric/emotional assessments before their standard clinical evaluation. Likewise, psychiatrists and clinical psychologists may need to conduct EEG/MRI scans and 24-h cardiac holter monitoring to consider depression as a neurocardiovascular disorder.”

## Data Availability

The original contributions presented in the study are included in the article/supplementary material, further inquiries can be directed to the corresponding author.
